# Evaluation of a peer-assisted, simulation-based clinical skills training program in Spain: a prospective single-group before-and-after study

**DOI:** 10.3352/jeehp.2026.23.15

**Published:** 2026-06-11

**Authors:** Maria López-Brotons, Sergio Javaloy-Ballestero, José-Manuel Ramos-Rincón

**Affiliations:** 1 Department of Clinical Medicine, School of Medicine, Miguel Hernández University of Elche, Alicante, Spain; 2 Technical Support Service for Teaching and Research, School of Medicine, Miguel Hernández University of Elche, Alicante, Spain; Hallym University, Korea

**Keywords:** Clinical competence, Personal satisfaction, Self-report, Undergraduate medical education, Spain

## Abstract

**Purpose:**

This study aimed to evaluate the implementation and evolution of a peer-assisted, simulation-based clinical skills program at Miguel Hernández University, Spain, focusing on medical student participation, perceptions, and perceived educational value.

**Methods:**

A prospective quasi-experimental pre–post study without a control group was conducted. Sessions were organized in small groups and led by senior student tutors under faculty supervision. Six workshops were offered during the first academic year and 8 during the second, with the addition of lumbar puncture and introductory clinical ultrasound. Knowledge acquisition was assessed using pre- and post-workshop questionnaires, and satisfaction was evaluated with Likert-scale surveys.

**Results:**

A total of 139 unique students participated across the 2 academic years, with 77 participants in 2023–2024 and 77 in 2024–2025; 15 students participated in both academic years. These participants generated 440 workshop attendances. After incomplete questionnaires were excluded, 425 paired pre- and post-workshop evaluations were analyzed. Students reported improved learning, with workshop-specific knowledge test scores increasing significantly from 6.1±2.6 to 8.7±1.6 (Δ=2.5; 95% confidence interval [CI], 2.29–2.71; Cohen’s d=1.14; P<0.001). Male students showed greater knowledge gain than female students (Δ=2.8 vs. 2.3; 95% CI, 2.17–3.43 vs. 1.91–2.69; Cohen’s d=1.17 vs. 1.15; P=0.034), and second-year students improved more than third-year students (Δ=2.9 vs. 2.1; 95% CI, 2.36–3.44 vs. 1.67–2.53; Cohen’s d=1.21 vs. 1.11; P=0.001). Satisfaction was high, with mean scores above 4/5.

**Conclusion:**

Clinical simulation combined with peer tutoring was feasible and well accepted in an undergraduate medical curriculum in Spain, achieving sustained participation over 2 academic years and consistently high satisfaction ratings. The program was associated with significant immediate improvements in workshop-specific knowledge test scores.

## Graphical abstract

[Fig f2-jeehp-23-15]

## Introduction

### Background

The acquisition of clinical skills is a fundamental component of undergraduate medical education. Traditionally, these competencies have been developed during clinical rotations with real patients. In recent years, clinical simulation has increasingly been incorporated as an educational tool that enables training in procedures and examination skills in a safe, controlled, and repeatable environment, thereby facilitating the integration of theoretical knowledge with clinical practice and improving student confidence [1].

At the same time, medical education has shifted toward more student-centered and participatory approaches. Among these, peer-assisted learning, or near-peer teaching, has emerged as a widely used strategy in the health sciences [2,3]. In this model, senior students facilitate the learning of junior students, promoting active engagement and collaborative learning. Previous studies have shown that this approach may benefit both learners and tutors by enhancing knowledge acquisition, communication skills, and teaching competencies [4,5].

This program was informed by principles of peer-assisted learning, particularly cognitive and social congruence between tutors and learners, as well as by experiential learning theory, which emphasizes learning through active experience, reflection, and application. These frameworks guided the workshop design, which focused on active participation, feedback, and repetition within a simulation-based environment.

The combination of clinical simulation and peer-assisted learning offers a valuable opportunity to expand practical training while optimizing available educational resources [6]. This approach is especially relevant in medical schools facing increasing student numbers and limited faculty availability, where access to individualized clinical training may be constrained [2,7].

In this context, the Department of Clinical Medicine at Miguel Hernández University developed a peer-assisted, simulation-based program to train second- and third-year medical students in clinical skills. The program was implemented over 2 academic years, progressively incorporating new workshops and methodological adaptations. This study was designed as a program evaluation to assess its implementation and perceived educational value.

### Objectives

The objective of this study was to describe the implementation and evolution of a peer-assisted, simulation-based clinical skills training program over 2 academic years and to evaluate students’ perceptions and perceived educational value. From a program-evaluation perspective, the study assessed implementation processes, workshop-specific knowledge test scores, and student satisfaction.

## Methods

### Ethics statement

The study was approved by the Committee on Ethics and Integrity in Research at Miguel Hernández University of Elche (TFG.GME.JMRR.MLB.241029). The requirement for informed consent was waived.

### Study design

This was a prospective, single-center, nonrandomized educational intervention evaluation using a 1-group pretest–posttest before-and-after design with repeated measures at the workshop-attendance level over 2 academic years. The study is reported in accordance with the Strengthening the Transparent Reporting of Evaluations with Nonrandomized Designs (TREND) statement [8].

### Setting

The study was conducted at the Clinical Simulation Area of Miguel Hernández University (ASCUMH), located in the Faculty of Medicine on the Sant Joan d’Alacant campus in Alicante, Spain. This teaching facility is equipped with low- and medium-fidelity simulators and specific infrastructure for training in clinical skills and basic procedures.

The peer-tutoring program was implemented over 2 consecutive academic years, 2023–2024 and 2024–2025, as a complementary training activity within courses with practical content in the Bachelor of Medicine program. Recruitment and data collection took place from February 2024 to May 2024 for the 2023–2024 academic year and from February 2025 to May 2025 for the 2024–2025 academic year.

### Participants

The study population consisted of undergraduate medical students at Miguel Hernández University who were enrolled in courses that included clinical skills training.

Eligible participants were all second-year students enrolled in General Pathology and all third-year students enrolled in Integrated Workshops II during each academic year (eligible N=254 in 2023–2024 and N=258 in 2024–2025). Students were invited via course announcements and email, and workshop registration was managed through UMH Virtual Campus, a platform developed using Moodle (https://moodle.org/). The inclusion criterion was attendance at ≥1 workshop session. For the paired pre–post analysis, attendances were included only when both the pre- and post-workshop knowledge tests had been completed; attendances missing either assessment were excluded from paired analyses.

### Intervention

The intervention consisted of a peer-assisted, simulation-based program focused on physical examination skills and basic procedures. Workshops were conducted in small groups of 5 students to promote active learning.

Sessions were led by senior student tutors under faculty supervision. Tutors were selected based on academic performance, prior experience, and motivation and received structured training covering content, teaching procedures, and learning objectives. Tutor objectives included reinforcing clinical knowledge, communication skills, and teaching competencies.

Across the 2 academic years, 6 workshops were delivered in 2023–2024 and 8 in 2024–2025. Topics included cardiac and pulmonary auscultation, abdominal examination, rectal and breast examination, neurological examination, basic clinical skills, lumbar puncture, and introductory clinical ultrasound (Table 1).

Each workshop session lasted approximately 60 minutes and followed a standardized sequence: brief introduction, demonstration, supervised practice, and feedback. Sessions included faculty supervision and periodic feedback to tutors. The intended exposure was 1 session per workshop registration; students could register for multiple workshops. No incentives were provided for participation.

### Outcomes

The primary outcome was the change in knowledge test score, calculated as the post-workshop score minus the pre-workshop score for each workshop attendance. Secondary outcomes included workshop participation, expressed as the occupancy rate, and student satisfaction ratings.

Knowledge tests were workshop-specific multiple-choice questionnaires consisting of 5–11 items, each with 1 correct option, developed by faculty based on workshop learning objectives. These tests were intended to capture short-term knowledge acquisition immediately after the session and were not designed as fully validated psychometric instruments.

### Data sources/measurement

The program was evaluated using questionnaires administered to participating students before and after each workshop. These instruments enabled assessment of both acquired knowledge and students’ perceptions of the training activity (Supplement 1).

To assess knowledge acquisition, brief questionnaires were administered before and after each session. Knowledge acquisition was assessed using workshop-specific multiple-choice questionnaires developed by faculty members based on the learning objectives of each session. Each questionnaire consisted of 5 to 11 items, with 4 response options per question and a single correct answer. Each correct answer was scored as 1 point, with no penalty for incorrect responses, resulting in a total score ranging from 0 to the maximum number of items. The same questionnaire was administered before and after each workshop to allow direct comparison of knowledge acquisition. Higher scores indicated greater knowledge of the specific clinical skill addressed in the session. Improvement in knowledge acquisition was defined as the difference between post-workshop and pre-workshop scores, with positive values reflecting learning gains associated with the intervention. These instruments were designed for formative assessment of immediate learning and were not intended as fully validated psychometric tools.

Questionnaires with incomplete data, defined as missing either the pre- or post-workshop assessment, were excluded from the paired analysis because calculation of knowledge improvement required both measurements. Missing data were assumed to be missing at random because non-completion was primarily related to logistical factors, such as absence at the time of questionnaire administration, rather than participant characteristics or performance.

At the end of each workshop, students also completed a satisfaction survey designed to gather their perceptions of the workshop’s usefulness, the clarity of the explanations, the organization of the session, and the effectiveness of peer tutoring as a teaching methodology. During the 2023–2024 academic year, the survey included 9 questions with responses based on a 1-to-5 Likert scale. In the 2024–2025 academic year, the questionnaire was updated, expanded, and validated to include 12 questions while maintaining a 1-to-5 Likert-type rating scale (Supplement 2). The updated questionnaire incorporated additional items based on a previously validated survey on clinical simulation and student satisfaction [9], ensuring content validity. Despite the difference in the number of items between academic years, the core domains—usefulness, clarity, organization, and perceived effectiveness—and the response scale remained consistent. Therefore, results were pooled for the overall analysis, while comparisons by academic year were also performed to account for potential differences related to modification of the instrument.

### Bias

Potential biases included self-selection because participation was voluntary, Hawthorne or novelty effects, testing effects from repeating the same pre- and post-workshop questionnaire, and clustering because students attended multiple workshops. To reduce bias, all eligible students were invited, workshops followed a standardized protocol with trained tutors and faculty supervision, knowledge tests were objectively scored, and surveys were anonymous. Missing pre–post data were excluded, and analyses were designed to adjust for repeated measures. Residual confounding remains because there was no control group.

### Study size

No formal sample size calculation was performed because this study was designed as a program evaluation. All eligible students were invited, and all available workshop attendances during the study period were included.

### Assignment method

Because this was a voluntary single-group before-and-after evaluation, no random assignment was used, and no comparison group was included. All eligible students were invited to attend workshops, and outcomes were assessed within participants, comparing pre- and post-workshop scores for each workshop attendance.

### Blinding

Participants and tutors were not blinded to the intervention. Knowledge tests were scored using an objective answer key; therefore, assessor blinding was not applicable.

### Statistical methods

Data from both academic years were pooled, including an indicator variable for academic year. Quantitative variables were summarized as means and standard deviations (SDs) or as medians and interquartile ranges, depending on distribution. Normality was assessed using the Shapiro-Wilk test. Categorical variables were expressed as frequencies and percentages.

Group comparisons for workshop attendance were performed using the Mann-Whitney U test. Pre–post comparisons were analyzed using the paired Student t-test. Categorical variables were compared using the chi-square test or Fisher exact test.

For pre–post analyses, mean differences (Δ), 95% confidence intervals (CIs), and Cohen’s d effect sizes were calculated. A P-value <0.05 was considered statistically significant. Analyses were performed using IBM SPSS Statistics ver. 25.0 (IBM Corp.).

## Results

### Participants

**Fig. 1.**
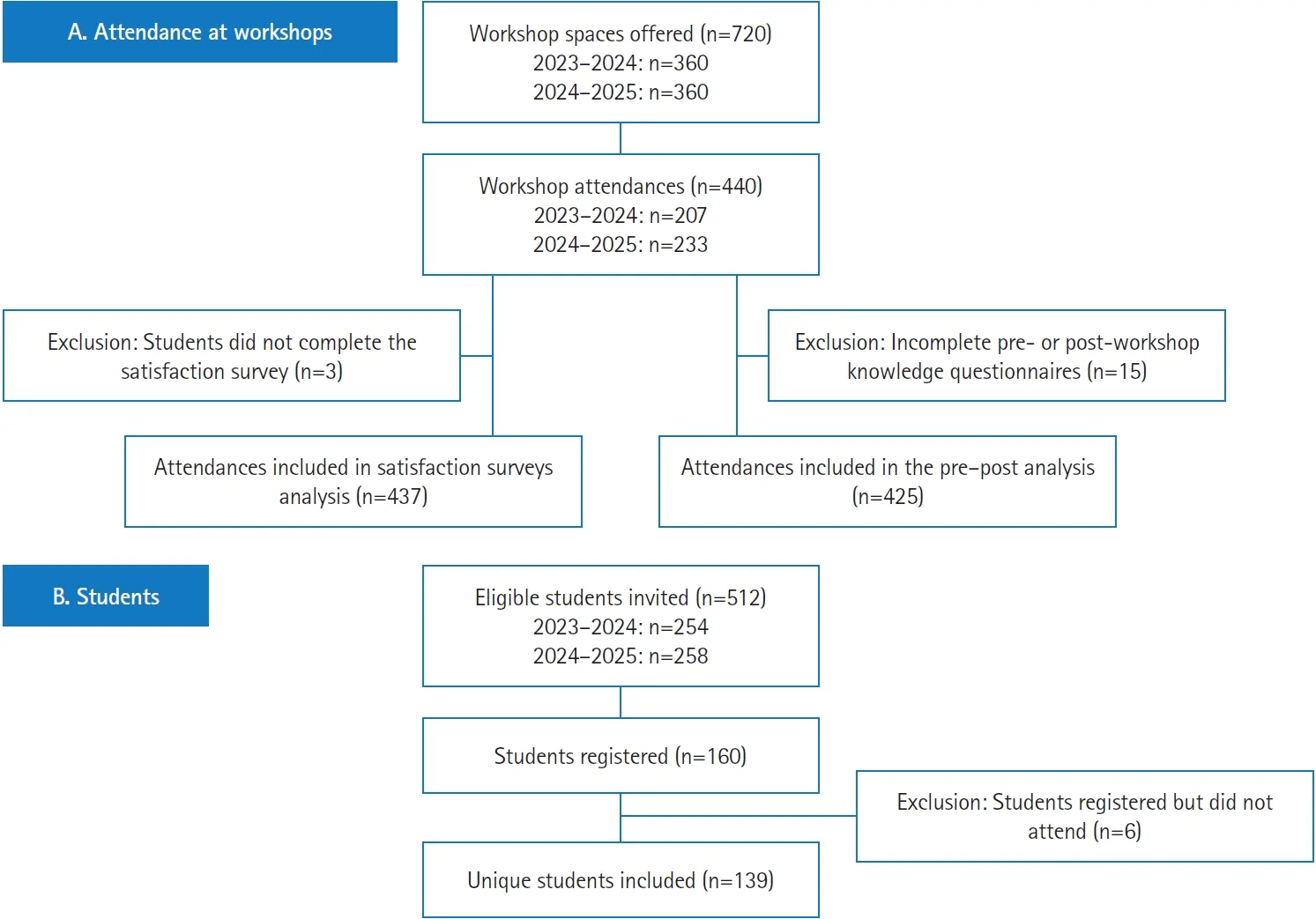
Flow chart.

The participant flow diagram is presented in Fig. 1. Of the 254 and 258 students eligible during the 2023–2024 and 2024–2025 academic years, respectively, 77 students participated in each academic year. Fifteen students participated in both academic years; therefore, these 154 academic-year participant records corresponded to 139 unique students. Across both academic years, 720 workshop places were offered, resulting in 440 attendances. Of these, 425 attendances were included in the pre–post knowledge analysis after 15 with incomplete assessments were excluded. Satisfaction survey responses were available for 437 attendances because 3 students did not complete the satisfaction survey.

During the 2023–2024 academic year, 6 simulation-based clinical skills workshops were offered, with 207 attendees and an occupancy rate of 57.5%. In the 2024–2025 academic year, 8 workshops were offered, with 233 attendees and an occupancy rate of 64.7%. Overall, 720 places were offered during the 2 academic years, with 440 attendances, representing an overall occupancy rate of 61.1%. The workshop with the highest overall participation was the neurological examination workshop (71.8%) (Dataset 1). Details of participation in each workshop are presented in Table 2.

Across the 2 academic years, 77 students participated in each academic year. Fifteen students participated in both academic years, resulting in 154 academic-year participant records corresponding to 139 unique students. Some students who participated during their second year in 2023–2024 participated again during their third year in 2024–2025. Women predominated, accounting for 64.3% of participants. In the 2024–2025 academic year, men attended more workshops than women (median, 6 vs. 2 attendances; P=0.003), while in 2023–2024 the differences were smaller. Regarding academic year of study, participation in 2023–2024 was predominantly among second-year students, whereas participation in 2024–2025 was higher among third-year students. In the 2024–2025 academic year, second-year students attended more workshops than third-year students (median, 4 vs. 1.5 attendances; P=0.003). Details of these characteristics are presented in Table 3.

### Learning acquired after the workshops

During the study period, 440 workshop attendances were recorded, as each student could participate in more than 1 workshop. Fifteen questionnaires were not completed correctly, mainly because either the pre- or post-workshop assessment was missing, and were therefore excluded (Fig. 1). Thus, 425 complete paired questionnaires were included in the final analysis. Consequently, the learning analysis was performed on 425 pre-workshop assessments and 425 post-workshop assessments (Dataset 2).

Overall, knowledge increased significantly after participation in the workshops (P<0.001) (Table 4). The overall mean score rose from 6.1 (SD, 2.6) before the workshop to 8.7 (SD, 1.6) after the workshop, with an average improvement of 2.5 points. This improvement was associated with a large effect size (Cohen’s d=1.14) and a 95% CI of 2.29–2.71.

In the 2023–2024 academic year, the largest increase in scores was observed in the pulmonary auscultation workshop (Δ=3.6 points), whereas the smallest increase was recorded in the rectal and breast examination workshop (Δ=1.8 points). In the 2024–2025 academic year, the largest increase was observed in the introduction to clinical ultrasound workshop (Δ=3.3 points), whereas the smallest increase was again observed in the rectal and breast examination workshop (Δ=2.1 points). Detailed results by academic year are presented in Supplement 3.

In the overall analysis of both academic years, the greatest increase in knowledge was observed in the pulmonary auscultation workshop (Δ=3.1 points; Cohen’s d=1.41; 95% CI, 2.48–3.72), followed by the neurological examination workshop (Δ=2.9 points; Cohen’s d=1.38; 95% CI, 2.40–3.40). The smallest increase was observed in the rectal and breast examination workshop (Δ=1.9 points) (Table 4).

In the analysis by gender, male students showed a greater gain in knowledge than female students (Δ=2.8 vs. 2.3 points; Cohen’s d=1.17 vs. 1.15; 95% CI, 2.17–3.43 vs. 1.91–2.69; P=0.034). Similarly, second-year students showed greater improvement than third-year students (Δ=2.9 vs. 2.1 points; Cohen’s d=1.21 vs. 1.11; 95% CI, 2.36–3.44 vs. 1.67–2.53; P=0.001).

### Satisfaction survey

After completing each workshop, students completed a satisfaction survey. Satisfaction survey responses were available for 437 attendances because 3 students did not complete the survey (Fig. 1). In the 2023–2024 academic year, the questionnaire included 9 items; in the 2024–2025 academic year, it was expanded to 12 items, while a 1-to-5 Likert-type rating scale was maintained in both years (Dataset 3).

In both academic years, the workshops received very positive ratings, with average scores above 4 points for all evaluated items (Table 5). In the 2023–2024 academic year, the most highly rated aspects were the usefulness of the training method and the student facilitator’s contribution to learning (mean, 4.9). In the 2024–2025 academic year, the usefulness of simulation for learning, improvement of technical skills, and integration of theoretical knowledge with clinical practice were particularly highly rated (mean, 4.9–5.0).

## Discussion

### Key results

This study suggests that a peer-assisted, simulation-based clinical skills program was positively perceived by students and may support their learning experience. This study evaluated the implementation of clinical skills workshops based on clinical simulation and peer tutoring for second- and third-year medical students. The results showed a significant improvement in workshop-specific knowledge test scores following workshop participation, together with high levels of student satisfaction. These findings provide preliminary evidence of positive student perceptions and perceived learning benefits associated with the program.

The observed effect sizes were large across all comparisons, suggesting substantial improvement in workshop-specific knowledge test scores immediately after the workshops. However, because these assessments measured only immediate knowledge acquisition using workshop-specific multiple-choice tests and were not validated psychometric instruments, the findings should not be interpreted as evidence of long-term knowledge retention or clinical competence.

### Interpretation

From an educational perspective, these findings suggest that combining simulation with peer-assisted learning may enhance student engagement and perceived learning by providing a structured, interactive, and low-hierarchy environment [2,3]. The active participation and repeated practice inherent in this approach may explain the observed improvements in perceived knowledge and confidence [2,3].

The greater gains observed among second-year students may reflect their lower baseline exposure to clinical skills, allowing a larger margin for improvement. Similarly, the high levels of satisfaction suggest that this model may be particularly well accepted in the early stages of clinical training, when students benefit from guided practice in a safe environment.

However, because the outcomes were based on self-reported measures and short-term assessments, these findings should be interpreted cautiously and as indicative of perceived learning rather than actual performance gains.

### Comparison with previous studies

Clinical simulation is widely used in medical education and has been associated with improved learning and skill acquisition [1,10,11]. Peer-assisted learning has also been shown to support student engagement and knowledge development [2,3,12].

Our findings are consistent with previous studies suggesting that combining simulation and peer tutoring may be beneficial, particularly for perceived learning and student experience [13,14]. This approach may also contribute to a more collaborative learning environment and support the development of communication and teaching skills among tutors.

### Limitations

This study has several limitations. First, voluntary participation may have introduced selection bias, as more motivated students may have been overrepresented. Second, the absence of a control group limits causal interpretation. Third, outcomes included workshop-specific knowledge test scores and self-reported satisfaction measures and were assessed only in the short term, without evaluation of long-term knowledge retention or transfer to clinical practice.

Additionally, analyses were performed at the level of workshop attendances rather than individual participants, which may have introduced clustering effects. Finally, the single-institution setting may limit generalizability.

### Generalizability

Despite these limitations, the study has several strengths. First, it describes the implementation of an innovative educational experience that combines clinical simulation and peer learning in undergraduate medical education. The literature suggests that integrating these methodologies may promote active learning, enhance student engagement, and support the acquisition of clinical skills in a safe environment [1,2,11,13]. Second, the analysis of 2 consecutive academic years allowed changes in student engagement and learning over time to be observed, providing a longitudinal perspective on the educational intervention. Finally, the results reinforce existing evidence regarding the potential for incorporating students themselves as learning facilitators within clinical simulation programs.

### Suggestions for further studies

Future research should evaluate the impact of this type of intervention not only on theoretical knowledge but also on the acquisition of practical clinical skills and on knowledge retention in the medium and long term. Studies with experimental designs that include control groups or comparisons with traditional teaching methods would also provide more robust evidence regarding the effectiveness of peer tutoring in clinical training [11].

### Conclusions

Overall, the integration of clinical simulation and peer tutoring represents a promising educational approach for clinical skills training. The program was associated with improvements in self-reported knowledge and high levels of satisfaction. However, the findings should be interpreted as reflecting perceived rather than actual effectiveness. Further research incorporating objective outcomes is warranted.

## Figures and Tables

**Figure f2-jeehp-23-15:**
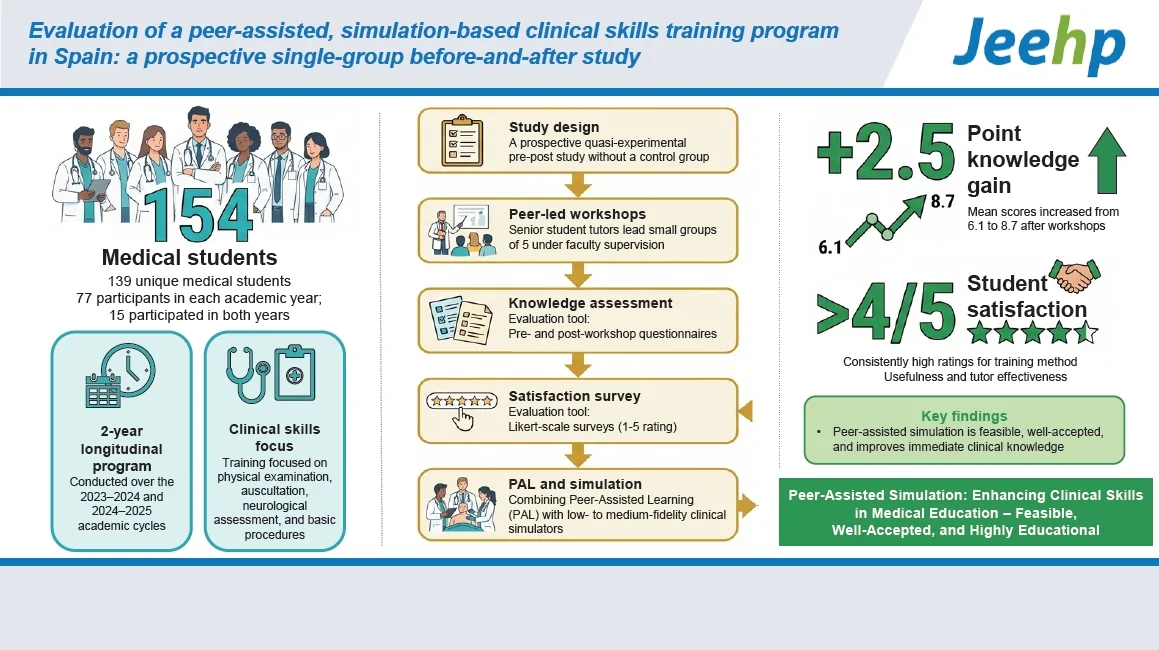


**Table 1.** Content of the peer-tutored clinical skills workshops

Workshop	Academic year(s) implemented	Main content
Cardiac auscultation	2023–2024/2024–2025	Identification of auscultation sites and recognition of normal and pathological heart sounds using simulation manikins. In the second year, clinical cases were incorporated for clinical correlation.
Pulmonary auscultation	2023–2024/2024–2025	Training in thoracic auscultation techniques and identification of normal and abnormal breath sounds using simulators.
Abdominal examination	2023–2024/2024–2025	Complete abdominal physical examination (inspection, auscultation, palpation, and percussion) with practice on simulators and among students.
Rectal and breast examination	2023–2024/2024–2025	Practice of digital rectal examination and breast examination using simulators with different clinical scenarios.
Neurological examination	2023–2024/2024–2025	Assessment of cranial nerves, reflexes, sensation, strength, gait, balance, and meningeal signs.
Basic clinical skills	2023–2024/2024–2025	Taking vital signs and performing electrocardiograms on students.
Lumbar puncture	2024–2025	Training in indications, materials, and lumbar puncture technique using a simulator.
Introduction to clinical ultrasound	2024–2025	Introduction to ultrasound machine operation and recognition of anatomical structures using a portable ultrasound device.

**Table 2.** Occupancy rate of peer-tutored examination and skills workshops by academic year

Workshop	Total participation rate	2023–2024 occupancy rate	2024–2025 occupancy rate	P-value
Cardiac auscultation	60.4 (58/96)	45 (27/60)	86.1 (31/36)	**<0.001**
Pulmonary auscultation	50 (48/96)	41.6 (25/60)	63.8 (23/36)	**0.035**
Abdominal examination	61.4 (59/96)	65 (39/60)	55.5 (20/36)	0.34
Rectal and breast examination	67.7 (65/96)	63.3 (38/60)	75 (27/36)	0.23
Neurological examination	71.8 (69/96)	73.3 (44/60)	69.4 (25/36)	0.68
Basic clinical skills	59.3 (57/96)	56.6 (34/60)	63.8 (23/36)	0.39
Lumbar puncture	48.6 (35/72)	-	48.6 (35/72)	-
Introduction to clinical ultrasound	66.6 (48/72)	-	66.6 (48/72)	-
Total	61.1 (440/720)	57.5 (207/360)	64.7 (233/360)	**0.045**

Values are presented as % (number of attendees/number of available spots). Statistically significant results are marked in bold.

**Table 3.** Characteristics of participating students and number of attendances at peer-tutored clinical skills workshops

Characteristic	2023–2024	2024–2025	Total
Participating students			
Total	77	77	154
Gender			
Male	31 (40.3)	24 (31.2)	55 (35.7)
Female	46 (59.7)	53 (68.8)	99 (64.3)
P-value	0.35		
Academic year			
Second	42 (54.5)	35 (45.5)	77 (50.0)
Third	35 (45.5)	42 (54.5)	77 (50.0)
P-value	0.12		
No. of workshop attendances			
Total	2 (1–5)	2 (1–5)	2 (1–5)
Gender			
Male	2 (1–4)	6 (4.5–8)	4 (1–6)
Female	2.5 (1–5)	2 (1–2)	2 (1–4)
P-value	0.44	0.003	0.007
Academic year			
Second	2 (1–4)	4 (2–6)	3 (1–5)
Third	2 (1–6)	1.5 (1–2)	2 (1–5)
P-value	0.37	0.003	0.12

Values are presented as number (%) or median (interquartile range) unless otherwise stated. The number of participating students is presented by academic year. The total of 154 represents the sum of participants counted separately in each academic year. Because 15 students participated in both academic years, these 154 academic-year participant records correspond to 139 unique students.

**Table 4.** Knowledge of clinical examination skills before and after peer-tutoring workshops

Variable	Pre-workshop mean±SD	Post-workshop mean±SD	Δ Mean±SD	95% CI	Cohen’s d	P-value
Total	6.1±2.6	8.7±1.6	2.5±2.2	2.29–2.71	1.14	<0.001
Workshops						
Cardiac auscultation	6.6±3.0	9.0±1.7	2.5±2.7	1.80–3.20	0.93	<0.001
Pulmonary auscultation	4.3±2.1	7.5±1.9	3.1±2.2	2.48–3.72	1.41	<0.001
Abdominal examination	5.9±2.5	8.3±1.7	2.4±2.1	1.87–2.93	1.14	<0.001
Rectal and breast examination	6.8±2.2	8.8±1.4	1.9±1.8	1.46–2.34	1.06	<0.001
Neurological examination	5.8±2.6	8.8±1.4	2.9±2.1	2.40–3.40	1.38	<0.001
Basic clinical skills	7.1±2.3	9.1±1.2	2.0±1.9	1.51–2.49	1.05	<0.001
Lumbar puncture	6.3±2.6	8.8±1.1	2.5±2.6	1.64–3.36	0.96	<0.001
Introduction to clinical ultrasound	5.2±2.4	8.5±1.5	3.3±2.1	2.71–3.89	1.57	<0.001
Gender						
Male	5.7±2.6	8.6±1.6	2.8±2.4	2.17–3.43	1.17	0.034
Female	6.5±2.5	8.7±1.6	2.3±2.0	1.91–2.69	1.15	-
Academic year						
Second	5.4±2.7	8.3±1.8	2.9±2.4	2.36–3.44	1.21	0.001
Third	6.7±2.3	8.9±1.3	2.1±1.9	1.67–2.53	1.11	-

Δ indicates the difference between post- and pre-workshop scores. Effect sizes (Cohen’s d) were calculated as the mean difference divided by the SD of the differences. SD, standard deviation; CI, confidence interval.

**Table 5.** Evaluation of peer-tutored clinical skills workshops by academic year

Evaluated item	2023–2024	2024–2025
Perceived improvement in learning	4.8±0.5	4.9±0.3
Workshop met expectations	4.8±0.4	-
Usefulness of audiovisual material	4.8±0.5	-
Usefulness of the training method	4.9±0.4	4.9±0.2
Workshop booking system	4.7±0.7	-
Simulation room setup	4.8±0.4	-
Simulation facilities and resources	4.8±0.5	-
Simulator quality	4.6±0.7	-
Contribution of the student facilitator	4.9±0.2	4.8±0.5
Realism of the simulation scenarios	-	4.7±0.6
Improvement of technical skills	-	4.9±0.3
Appropriateness of the simulated environment	-	4.8±0.4
Improved safety and confidence	-	4.9±0.4
Integration of theory and practice	-	4.9±0.4
Motivation to learn	-	4.9±0.4
Workshop duration	-	4.8±0.3
Usefulness of peer tutoring	-	4.9±0.3
Overall satisfaction with the experience	-	5.0±0.2

Values are presented as mean±standard deviation. Likert scale from 1 (lowest) to 5 (highest).

## Footnotes

**Data availability**
Data files are available from https://doi.org/10.7910/DVN/ONMKTM Dataset 1. Workshop attendance.

Dataset 2. Learning pre–post.

Dataset 3. Satisfaction survey.

## Supplementary materials

Supplement 1. Student pre- and post-workshop questionnaires in English and Spanish.

Supplement 2. Student satisfaction survey form.

Supplement 3. Comparison of knowledge levels before and after the peer-tutored clinical examination workshops, by workshop type, gender, and academic year.

Supplement 4. Audio recording of the abstract.
