# Supplementary material for: Evaluation of a peer-assisted, simulation-based clinical skills training program in Spain: a prospective single-group before-and-after study
Source: J Educ Eval Health Prof. 2026 Jun 11;23:15. doi: 10.3352/jeehp.2026.23.15 (PMC13506240; doi:10.3352/jeehp.2026.23.15)
Supplement: Supplementary file 1 [file jeehp-23-15-suppl1.docx]

## **Supplement 1.**

## **Student Pre- and Post-Workshop Questionaries**

The following questions appear on all questionnaires:

Select the grade you are in: you must select from 1st to 6th grade.

Select your gender:

-Female

-Male

-I’d rather not say

Have you attended a workshop before?

Yes, this same workshop.

Yes, another workshop.

No, never.

Enter your age

Select your assigned number (answers range from 1 to 18)

Have you reviewed the pre-workshop materials?

-No

-Yes

-Partially

- **WORKSHOP 1 - CARDIAC AUSCULTATION**

1- What is the aortic focus?
a) Green dot
b) Orange dot
c) **Purple dot**
d) White dot
2-Which one is Erb's focus?
a) Green dot
b) Orange dot
c) Purple dot
d) **White dot**
3- What type of murmur or noise is it?
a) Diastolic murmur
b) **Early systolic murmur**
c) Holosystolic murmur
d) Opening click
4- What type of murmur or sound is it?
a) Diastolic murmur
b) Early systolic murmur
c) Holosystolic murmur
d) **Opening click**
5- What type of murmur or sound is it?
a) Diastolic murmur
b) Early systolic murmur
c) **Holosystolic murmur**
d) Opening click
6- What type of murmur or sound is it?
a)**Diastolic murmur**
b) Early systolic murmur
c) Holosystolic murmur
d) Opening click

- **WORKSHOP 2—LUNG AUSCULTATION**

1- What is the normal respiratory rate?
a) 10–16 breaths per minute
b) 12–22 breaths per minute
c) 10–20 breaths per minute
d) **12–20 breaths per minute**
2- Regarding pulmonary auscultation, select the INCORRECT option.
a) Pulmonary auscultation is possible from both the anterior and posterior aspects of the chest.
b) **Both hemithoraces should be auscultated and compared, although this is not essential.**
c) The normal sound is vesicular breath sounds.
d) The lung bases are best auscultated from the posterior aspect of the chest.
3- Regarding pathological sounds, select the CORRECT option
a) Stridor is primarily caused by airway obstruction at the bronchial level
b) Wheezes are wet rales.
c) **Wet rales are classified as coarse, fine, or very fine.**
d) All of the above are false
4- Regarding pleural friction, select the CORRECT answer
a) Pleural friction is caused by friction between the parietal and visceral pleura
b) The sound heard on auscultation is coarse
c) It is characteristic of pleurisy
d) **All are correct**
5- Regarding asthma, select the CORRECT answer.
a) The auscultation of dry rales, specifically rhonchi, is characteristic.
b) The auscultation of crackles or wet rales is characteristic.
c) **It is characteristic to hear dry rales on auscultation, specifically wheezing.**
d) The first and third options are correct.
6- Regarding pneumonia, select the CORRECT answer.
a) Increased breath sounds are heard.
b) Diminished breath sounds are heard.
c) The first and third options are correct.
d) **The second and third options are correct.**
7- Regarding the following sound, select the CORRECT option. [Sound 1](https://drive.google.com/file/d/1gmxboCyMnCRc1NorVRuCCO5QImy_LL-b/view?usp=drive_link)
a) Stridor
b) **Sibilants**
c) Fine crackles
d) Rhonchi
8- For the following sound, select the CORRECT option. [Sound 2](https://drive.google.com/file/d/1pt1k5YbWHINAbtBeCeTasIaPCkScyWCE/view?usp=drive_link)
a) Stridor
b) Wheezing
c) Fine crackles
d) **Rhonchi**
9- For the following sound, select the CORRECT option [Sound 3](https://drive.google.com/file/d/1wG-TWxL3TKIavnJQ4Hz_6pHo19mr1r3q/view?usp=drive_link)
a) Stridor
b) Wheezing
c) **Fine crackles**
d) Rhonchi
10- For the following sound, select the CORRECT option [Sound 4](https://drive.google.com/file/d/1jw-vD74A5cCTS9HG3qyRhVU7nVEQYFdX/view?usp=drive_link)
a) Stridor
b) Wheezing
c) Fine crackles
d) **Coarse crackles**
11- For the following sound, select the CORRECT option. [Sound 5](https://drive.google.com/file/d/1vUVHTorYHlvQAAuPNy8cvze4c1xaTibD/view?usp=drive_link)
a) **Stridor**
b) Wheezing
c) Fine crackles
d) Rhonchi

- **WORKSHOP 3 - ABDOMINAL EXAMINATION**

1- Select the CORRECT option
a) In the upper right quadrant, we could locate the spleen.
b) Abdominal auscultation allows us to listen only to abdominal sounds.
c) In the lower right quadrant, we locate the sigmoid colon.
d) **In the upper left quadrant, we locate the left lobe of the liver.**
2- Select the incorrect option
a) It is possible to auscultate a murmur from the renal arteries
b) **Regarding the abdominal examination, the order would be: inspection, percussion, palpation, and auscultation**
c) Abdominal palpation is divided into superficial and deep
d) All are incorrect
3- Regarding abdominal examination techniques, identify the INCORRECT answer.
a) A positive McBurney’s sign is suggestive of appendicitis.
b) **Murphy’s sign is performed in the left hypochondrium; if it is painful during the patient’s deep inspiration, it is positive.**
c) A positive Blumberg's sign is suggestive of peritoneal irritation.
d) A positive Blumberg’s sign is suggestive of peritonitis.
4- Regarding a patient presenting with ascites, select the CORRECT option
a) The shifting dullness sign is examined
b) The wave sign is present and always transmitted
c) The patient may present with the jellyfish head sign
d) **All of the above**
5- Regarding the following sound, select the CORRECT option. [Sound 1](https://drive.google.com/file/d/1xbf96DJ0ZL8YDpmP2_zp4GFNXGslkB3J/view?usp=drive_link)
a) **Borborygmi**
b) Metallic sounds
c) Diarrhea
d) Arterial murmur

- **WORKSHOP 4 - RECTAL AND BREAST EXAMINATION**

1- Regarding the digital rectal exam, select the INCORRECT option
a) It allows you to determine the size and consistency of the prostate
b) **It does not allow for the detection of proctological pathology**
c) It allows for the investigation of causes of gastrointestinal bleeding
d) There is no incorrect option
2- Regarding the digital rectal exam, select the INCORRECT answer
a) You must wear gloves and apply lubricant to your index finger.
b) The examination should be performed in the Douglas pouch.
c) The examination positions are: lateral decubitus, prone decubitus, and quadruped
d) **After completing the digital rectal exam, the glove is immediately removed and discarded**
3- Regarding prostate pathology, select the INCORRECT option
a) **In unilateral prostatic hypertrophy, only one hypertrophic lobe is found, and it is usually malignant**
b) In benign prostatic hypertrophy, the prostate is usually larger than normal
c) Unilateral prostate carcinoma is malignant and is found in only one prostate lobe
d) There is no incorrect option
4- Regarding breast examination, identify the INCORRECT answer:
a) Look for asymmetries and epithelial retractions.
b) **Only the breasts should be examined.**
c) The nipple must be squeezed, or the patient must be asked to squeeze her nipple.
d) Dermatological changes in the skin of the breast are suggestive of breast pathology.
5- Identify the INCORRECT answer regarding breast pathology
a) Lesions can be benign, malignant, or lymphadenopathies.
b) **A fibroadenoma is an irregular lesion and, therefore, malignant in nature.**
c) Malignant lesions are irregular in size, firm, and generally painless.
d) A fibrocystic breast is one that is irregular in consistency, hard, and enlarged.
6- Indicate which of the following is NOT a warning sign of breast cancer.
a) Skin changes
b) Change in breast size and shape.
c) Sudden nipple discharge
d) **All are warning signs**

- **WORKSHOP 5 - NEUROLOGICAL EXAMINATION**

1- Regarding the examination of the cranial nerves, select the INCORRECT answer
a) **One of the tests involves assessing facial sensation, which corresponds to the facial nerve**
b) Eye movements and the pupillary reflex are examined
c) Visual acuity is tested using a visual acuity chart, which corresponds to the optic nerve
d) The fundus is examined
2- Regarding the examination of the cranial nerves, select the INCORRECT option
a) The patient is asked to smile, show their teeth, and furrow their brow, corresponding to the facial nerve
b) Hearing is tested with a tuning fork
c) **The patient is asked to shrug their shoulders to assess the vagus nerve**
d) The patient is asked to stick out and move their tongue to assess the hypoglossal nerve
3- Regarding the examination of muscle strength, select the INCORRECT option
a) The limbs are passively moved to assess muscle tone
b) To assess hand strength, we ask the patient to squeeze our hand
c) **The patient is asked to hold their arms elevated; in cases of claudication, strength is preserved**
d) In the lower extremities, to assess proximal strength, the patient is asked to lift their legs and prevent the physician from lowering them.
4- Regarding reflex testing, select the INCORRECT option
a) In the patellar reflex, the quadriceps tendon is tapped
b) In the triceps reflex, there is contraction of the triceps and extension of the arm
c) **The plantar reflex is positive when flexion occurs, commonly known as a positive Babinski sign**
d) In the chin reflex, the skin of the chin is stimulated with a reflex hammer, causing contraction of the lower lip and chin
5- Regarding sensation, select the INCORRECT answer
a) Cortical sensory function allows the patient to recognize an object
b) A cold tuning fork is used to test thermal sensitivity
c) **Vibratory sensation cannot be tested in the clinic**
d) A cotton swab is used to test fine sensation
6- Regarding the assessment of coordination and gait, select the INCORRECT answer
a) Gait is assessed by asking the patient to walk normally, on tiptoes, on heels, and in tandem
b) Both static and dynamic coordination are assessed
c) The Romberg test is part of static coordination
d) **Dynamic coordination includes the Miller-Fisher test, in which the patient is asked to touch the doctor’s index finger with their own**

- **WORKSHOP 6 - SKILLS**

1- Regarding blood pressure measurement, select the INCORRECT answer
a) **The sphygmomanometer should be placed so that it aligns with the ulnar artery.**
b) Ensure that the cuff is closed
c) It can be taken using the radial pulse or a stethoscope
d) An aneroid or automatic sphygmomanometer can be used
2- Regarding blood pressure measurement, select the INCORRECT option
a) The pressure is increased with the sphygmomanometer to over 200 mmHg in order to take the blood pressure.
b) **The first sound heard is the diastolic blood pressure**
c) To remove the cuff, the valve must be opened completely
d) An automatic sphygmomanometer allows us to measure blood pressure while the patient is fully clothed
3- Regarding pulse measurement, select the INCORRECT option
a) It is usually taken by placing the index and middle fingers over the artery in question
b) **The carotid pulse should be taken at the same time to detect irregularities**
c) The radial artery is located on the inner side of the wrist, near the first metacarpal bone
d) You usually count the beats in 15 seconds and then multiply by 4
4- Select the INCORRECT answer
a) Respiratory rate can be measured by counting the number of times a patient’s chest or abdomen rises in 15 seconds and then multiplying by 4
b) The pulse oximeter is usually placed on the index finger
c) **The pulse oximeter is a very effective method, and having painted nails does not lead to errors**
d) The pulse oximeter provides the respiratory rate and heart rate; it is important to wait for the reading to stabilize
5- Select the INCORRECT answer regarding the electrocardiogram
a) It is used to record the heart’s electrical activity from the skin
b) **It only allows us to determine the rhythm and check how the heart’s electrical activity is functioning**
c) Electrodes are used
d) The electrocardiogram displays the following waves: P, Q, R, S, T, and U
6- Select the INCORRECT answer regarding the electrocardiogram
a) **Electrodes are always placed on the chest**
b) It can determine whether one heart chamber is larger than another
c) In ST-segment elevation myocardial infarction (STEMI), there is ST-segment elevation
d) Atrial fibrillation is a type of arrhythmia

- **WORKSHOP 7 - LUMBAR PUNCTURE**

1- Regarding the materials needed to perform a lumbar puncture, select the incorrect option
a) It is essential to use: sterile gloves, two sterile drapes, and a gown
b) The tubes for collecting CSF must be sterile
c) **Local anesthesia is loaded and administered using a subcutaneous needle**
d) The spinal puncture needle is of a large gauge
2- Regarding the procedure, select the correct answer
a) The patient is positioned sitting or in the lateral decubitus position with legs extended
b) Local anesthesia is administered intramuscularly
c) First, we wash our hands, put on sterile gloves, place the sterile drapes, and apply the antiseptic to the back in a linear pattern
d) **First, we wash our hands, put on sterile gloves, place sterile drapes, and apply the antiseptic to the back in a circular motion from the inside out**
3- When performing a lumbar puncture, what is the correct procedure once cerebrospinal fluid (CSF) begins to flow out?
a) Collect all available CSF for analysis
b) **Collect approximately 10 drops per tube into sterile tubes**
c) Aspirate the CSF with a syringe to speed up the process
d) Stop the puncture and remove the needle immediatel
4- Which of the following statements about lumbar punctures is incorrect?
a) You must check that the needle safety mechanism is working properly before starting the puncture.
b) **The bevel of the needle should be oriented downward to facilitate the flow of CSF**
c) After removing the needle, pressure should be applied to the puncture site to prevent complications
d) It is important to disinfect and protect the area after completing the procedure
5- What is the proper procedure for locating the lumbar puncture site?
a) **Palpate the anterior superior iliac spine and draw a line to the spinous process at the same level**
b) Identify the T6-T7 thoracic level and perform the puncture at that site
c) Locate the spinous process of L1-L2 and perform the puncture at that point
d) Palpate the iliac crest and perform the puncture directly at the most prominent vertebra

- **WORKSHOP 8 - INTRODUCTION TO CLINICAL ULTRASOUND**

1- Which of the following options correctly describes the function of the "Freeze"  button on an ultrasound machine?
a) It allows you to select the appropriate ultrasound probe for the study
b) It activates color Doppler to assess blood flow
c) **It freezes the image on the screen for later analysis**
d) It allows you to use pulsed Doppler to assess blood flow velocity
2- Which of the following statements about ultrasound probes is incorrect?
a) The convex probe is used in abdominal studies
b) The sector probe generates a triangular or fan-shaped image
c) The linear probe is ideal for musculoskeletal and vascular studies
d) **The sector probe is used in gynecological studies**
3- Which of the following ultrasound maneuvers involves moving the probe along all four axes (forward, backward, and sideways)?
a) Rotation
b) **Translation**
c) Tilt
d) Oscillation
4- What is the main use of the transverse ultrasound section?
a) **To evaluate anatomical relationships between structures**
b) To visualize organs along their longitudinal axis
c) To obtain images of tissues from top to bottom
d) None of the above
5- Which of the following organs is isoechoic on an ultrasound?
a) Liver
b) Spleen
c) Pancreas
d) **All of the above**
6- Which of the following is an example of an anechoic structure on an ultrasound scan?
a) Kidney stones
b) Fibrosis
c) **Amniotic fluid**
d) Liver
