# Supplementary material for: Evaluation of a peer-assisted, simulation-based clinical skills training program in Spain: a prospective single-group before-and-after study
Source: J Educ Eval Health Prof. 2026 Jun 11;23:15. doi: 10.3352/jeehp.2026.23.15 (PMC13506240; doi:10.3352/jeehp.2026.23.15)
Supplement: Supplementary file 2 [file jeehp-23-15-suppl2.docx]

**Supplementary 2. Student Satisfaction Survey. 2023-2024**

1- Do you feel that your learning has improved after attending the self-study classroom? (Select from 1, no, to 5, yes, significantly)
2- Did the workshop meet your initial expectations? (Select from 1, no, to 5, yes, significantly)
3- Rate the usefulness of the Simuloteca’s audiovisual content for learning clinical skills. (Where 1 is not very useful and 5 is very useful)
4- How would you rate the usefulness of this training method for your academic development? (Where 1 is not very useful and 5 is very useful)
5- If you rated any item negatively, could you explain why?
6- How would you rate the workshop reservation system in relation to planning your visit? (1: not very useful and difficult to use; 5: very useful and easy to use)
7- How would you rate the suitability of the venue for the workshop? (1: poor, 5: excellent)
8- Did the facilities and available resources facilitate learning? (Where 1 means no, they were inadequate, and 5 means yes, completely)
9- Did the simulators used meet the requirements for effective training? (Where 1 means no, they were inadequate, and 5 means yes, absolutely)
10- Did the student facilitator contribute positively to your learning experience? (1 = No, it made the experience worse; 5 = Yes, absolutely)

**SATISFACTION SURVEY ACADEMIC YEAR 2024-2025**

1- Do you find simulation to be a useful teaching method for learning? (Select 1 for not very useful and 5 for very useful)
2- The scenarios in which the simulation takes place are realistic. (Select 1 for not very realistic and 5 for very realistic)
3- Do you feel that the simulation workshops have helped you improve your technical skills? (Select 1 for not very much and 5 for very much)
4- The simulated environment aligns with my theoretical knowledge. (Select 1 for “not very” and 5 for “very”)
5- Do you feel that your experience with the workshop has helped you improve your confidence and self-assurance? (Select 1 for not at all and 5 for very much)
6- Do you feel that the simulation has helped you integrate theoretical knowledge with practice? (Select 1 for not at all and 5 for very much)
7- The workshops with the simulator have motivated me to learn. (Select 1 for a little and 5 for a lot)
8- Do you think the workshop’s duration is appropriate? (Select 1 for “not at all” and 5 for “very much”)
9- Is the facilitator’s training appropriate for this learning experience? (Select 1 for “not at all appropriate” and 5 for “yes, completely appropriate”)
10- How would you rate the usefulness of peer tutoring (where a student from a higher grade tutors students from lower grades) for your academic development? (Select a score from 1 [not very useful] to 5 [very useful])
11- Do you feel that your learning has improved after using the self-study room? (Select 1 for “no” and 5 for “yes, significantly”)
12- Overall, the simulation experience has been satisfactory. (Select 1 for “no” and 5 for “yes, significantly”)
13- If you rated any item negatively, could you explain why?

## **- ENCUESTAS DE SATISFACCIÓN**

**ENCUESTA DE SATISFACCIÓN CURSO ACADÉMICO 2023-2024**

1. ¿Sientes que tu aprendizaje ha mejorado tras acudir al aula de autoaprendizaje? (Seleccione del 1, no, al 5, sí significativamente)
2. ¿El taller realizado ha cubierto tus expectativas iniciales? (Seleccione del 1, no, al 5, sí significativamente)
3. Califica la utilidad del contenido audiovisual de la Simuloteca para el aprendizaje de la habilidad clínica. (Siendo el 1 poco útil y el 5 muy útil)
4. ¿Cómo calificarías la utilidad de este método de formación para tu desarrollo académico? (Siendo el 1 poco útil y el 5 muy útil)
5. Si has valorado algún punto negativamente, ¿podrías justificarlo?
6. ¿Cómo valoras el sistema de reserva del taller en relación a la planificación de tu visita? (1 poco útil y difícil de usar, 5 muy útil y sencillo de usar)
7. ¿Cómo valoras la adecuación de la estancia para la realización del taller? (1 deficiente, 5 excelente)
8. ¿Las instalaciones y los recursos disponibles facilitaron el aprendizaje? (Siendo 1 no, eran inadecuadas, y 5 sí, totalmente)
9. ¿Los simuladores empleados cumplían con los requisitos para un entrenamiento efectivo? (Siendo 1 no, eran inadecuadas, y 5 sí, totalmente)
10. ¿La estudiante facilitadora contribuyó positivamente a tu experiencia de aprendizaje? (Siendo 1 no, empeoró la experiencia y 5 sí, totalmente)

**ENCUESTA DE SATISFACCIÓN CURSO ACADÉMICO 2024-2025**

1. ¿Le parece la simulación un método docente útil para el aprendizaje? (Seleccione siendo el 1 poco útil y el 5 muy útil)
2. Los escenarios donde se desarrolla la simulación son realistas. (Seleccione siendo el 1 poco y el 5 muy realistas)
3. ¿Considera que los talleres de simulación le han ayudado a mejora sus habilidades técnicas? (Seleccione siendo el 1 poco y el 5 mucho)
4. El entorno simulado se adapta a mis conocimientos teóricos. (Seleccione siendo el 1 poco y el 5 mucho)
5. ¿Considera que su experiencia con el taller le ha ayudado a mejorar su seguridad y confianza? (Seleccione siendo el 1 poco y el 5 mucho)
6. ¿Considera que la simulación le ha ayudado a integrar el conocimiento teórico con la práctica? (Seleccione siendo el 1 poco y el 5 mucho)
7. Los talleres con el simulador me han motivado a aprender. (Seleccione siendo el 1 poco y el 5 mucho)
8. ¿Le parece adecuada la duración del taller? (Seleccione siendo el 1 poco y el 5 mucho)
9. ¿La capacitación de la facilitadora es adecuada para esta experiencia de aprendizaje? (Seleccione siendo el 1 no es nada adecuada y el 5 sí, totalmente adecuada)
10. ¿Cómo calificarías la utilidad de la tutoría entre pares (alumno de un curso superior tutoriza a alumnos de cursos inferiores) para tu desarrollo académico? (Seleccione siendo el 1 poco útil y el 5 muy útil)
11. ¿Sientes que tu aprendizaje ha mejorado tras acudir al aula de autoaprendizaje? (Seleccione siendo el 1 no y el 5 sí, significativamente)
12. En general, la experiencia en simulación ha sido satisfactoria. (Seleccione siendo el 1 no y el 5 sí, significativamente)
13. Si has valorado algún punto negativamente, ¿podrías justificarlo?
