# Supplementary material for: Evaluation of a peer-assisted, simulation-based clinical skills training program in Spain: a prospective single-group before-and-after study
Source: J Educ Eval Health Prof. 2026 Jun 11;23:15. doi: 10.3352/jeehp.2026.23.15 (PMC13506240; doi:10.3352/jeehp.2026.23.15)
Supplement: Supplementary file 3 [file jeehp-23-15-suppl3.docx]

**Supplementary 3. Table 1S.** Comparison of knowledge levels before and after the peer-tutored clinical examination workshops, by workshop type, gender, and academic year.

|  | **Academic Year 2023-24** | | | **Academic Year 2024-25** | | | **Total** | | |
| --- | --- | --- | --- | --- | --- | --- | --- | --- | --- |
|  | **Pre-workshop**  **Me (SD)**  **Mdn (RIC)** | **Post-workshop**  **Wed (DE)**  **Mdn (RIC)** | △  **Wed (DE)** | **Pre-workshop**  **Me (DE)**  **Wed (RIC)** | **Post-workshop**  **Me (DE)**  **Mdn (RIC)** | △  **Wed (DE)** | **Pre-workshop**  **Me (DE)**  **Wed (RIC)** | **Post-workshop**  **Me (DE)**  **Mdn (RIC)** | △  **Wed (DE)** |
| **Total** | 6.4 (2.6)  6.7 (5–8.3) | 8.8 (1.6)  10 (8–10) | 2.4 (2.1) ‡ | 5.9 (2.6)  6.3 (3.8–8.3) | 8.6 (1.5)  8.3 (8.0–10) | 2.6 (2.3) | 6.1 (2.6)  6.7 (4.0–8.3) | 8.7 (1.6)  8.3 (8.0–10) | 2.5 (2.2) |
| **Workshop** |  |  |  |  |  |  |  |  |  |
| Cardiac auscultation | 7.3 (3.1)  8.3 (5.0–10.0) | 9.4 (1.5)  10 (10–10) | 2.8 (0.5) ‡ | 6.0 (2.9)  6.7 (3.3–8.3) | 8.7 (1.8)  10 (8.3–10) | 2.7 (2.7) | 6.6 (3.0)  6.7 (3.3–10) | 9.0 (1.7)  10 (8.3–10) | 2.5 (2.7) |
| Pulmonary auscultation | 3.8 (2.0)  3.1 (2.4–5.5) | 7.3 (2.1)  7.1 (6.2–9.0) | 3.6 (2.2) ‡ | 4.9 (2.1)  4.5 (3.6–6.3) | 7.6 (1.7)  7.7 (6.3–9.1) | 2.7 (2.1) | 4.3 (2.1)  4.2 (2.7–5.8) | 7.5 (1.9)  7.3 (6.4–9.1) | 3.1 (2.2) |
| Abdominal examination | 6.2 (2.3)  6.0 (4.0–8.0) | 8.5 (1.8)  8.0 (6.2–9.0) | 2.3 (2.0) ‡ | 5.6 (2.7)  6.0 (4.0–8.0) | 8.1 (1.5)  8.0 (8.0–10) | 2.5 (2.4) | 5.9 (2.5)  6.0 (4.0–8.0) | 8.3 (1.7)  8.0 (8.0–10) | 2.4 (2.1) |
| Rectal and breast examination | 7.1 (2.3)  7.5 (5.0–8.3) | 8.9 (1.2)  8.3 (8.3–10) | 1.8 (1.8) ‡ | 6.7 (2.2)  (6.7 (5.0–8.3) | 8.8 (1.7)  10 (8.3–10) | 2.1 (1.9) | 6.8 (2.2)  6.7 (5.0–8.3) | 8.8 (1.4)  9.2 (8.3–10) | 1.9 (1.8) |
| Neurological examination | 6.1 (2.6)  6.7 (3.3–8.3) | 9.0 (1.4)  10 (7.5–10) | 2.9 (1.9) ‡ | 5.5 (2.5)  5.0 (5.0–6.7) | 8.5 (1.4)  8.3 (8.3–10) | 3.0 (2.4) | 5.8 (2.6)  5.8 (3.3–8.3) | 8.8 (1.4)  9.2 (8.3–10) | 2.9 (2.1) |
| Skills Workshop | 7.6 (1.6)  7.5 (6.7–8.3) | 9.4 (1.0)  10 (9.3–10) | 1.9 (1.4) ‡ | 6.5 (2.9)  6.7 (5.0–8.3) | 8.7 (1.4)  8.3 (8.3–10) | 2.2 (2.4) | 7.1 (2.3)  6.7 (6.7–8.3) | 9.1 (1.2)  10 (8.3–10) | 2.0 (1.9) |
| Lumbar puncture | - | - |  | 6.3 (2.6)  6.7 (5.0–8.3) | 8.8 (1.1)  8.3 (8.3–10) | 2.5 (2.6) | 6.3 (2.6)  6.7 (5.0–8.3) | 8.8 (1.1)  8.3 (8.3–10) | 2.5 (2.6) |
| Introduction to Clinical Ultrasound | - | - |  | 5.2 (2.4)  5.0 (3.3–6.7) | 8.5 (1.5)  8.3 (8.3–10) | 3.3 (2.1) | 5.2 (2.4)  5.0 (3.3–6.7) | 8.5 (1.5)  8.3 (8.3–10) | 3.3 (2.1) |
| **P-value*** | **<0.001** | **<0.001** | **0.003** | **0.048** | **0.008** | 0.27 | **<0.001** | **<0.001** | **0.001** |
| **Gender** | | | | | | | | | |
| Male | 6.4 (2.6)  6.7 (4.2–8.3) | 8.9 (1.6)  10 (8.1–10) | 2.5 (2.1) ‡ | 5.3 (2.6)  5.0 (3.3–8.3) | 8.3 (1.5)  8.3 (8.0–10) | 3.0 (2.5) | 5.7 (2.6)  5.4 (3.3–8.3) | 8.6 (1.6)  8.3 (8.0–10) | 2.8 (2.4) |
| Women | 6.3 (2.6)  6.7 (5.0–8.3) | 8.7 (1.6)  10 (8.0–10) | 2.4 (2.1) ‡ | 6.6 (2.3)  6.7 (5.0–8.3) | 8.7 (1.5)  10 (8.3–10) | 2.1 (2.0) | 6.5 (2.5)  6.6 (5.0–8.3) | 8.7 (1.6)  10 (8.0–10) | 2.3 (2.0) |
| **P-value**** | 0.39 | 0.39 | 0.75 | **0.001** | 0.085 | **0.02** | **0.007** | 0.22 | **0.034** |
| **Year** | | | | | | | | | |
| Second | 5.8 (2.8)  6.0 (3.3–8.3) | 8.5 (1.9)  9.0 (8.9–10) | 2.8 (2.4) ‡ | 5.2 (2.7)  5.0 (3.3–6.7) | 8.3 (1.6)  9.3 (6.7–10) | 3.0 (2.5) | 5.4 (2.7)  5.0 (3.3–8.0) | 8.3 (1.8)  8.3 (76.7–10) | 2.9 (2.4) |
| Third | 6.8 (2.4)  6.7 (5–8.3) | 8.9 (1.4)  10 (8.1–10) | 2.2 (1.8) ‡ | 6.7 (2.1)  6.7 (5.0–8.3) | 8.9 (1.3)  8.7 (8.3–10) | 2.1 (1.9) | 6.7 (2.3)  6.7 (5.0–8.3) | 8.9 (1.3)  10 (8.3–10) | 2.1 (1.9) |
| **P-value**** | **0.004** | 0.39 | 0.09 | **<0.001** | **0.002** | **0.006** | **<0.001** | **0.001** | **0.001** |

Abbreviation: %: percentage, SD: standard deviation, IQR: interquartile range

The p-value for the degree of knowledge before and after the workshop was calculated using the nonparametric Kruskal-Wallis test* and the Mann-Whitney U test**.

The p-value for the improvement in knowledge before and after the workshop was calculated using the repeated-measures Student’s t-test‡
